# Impact of SARS-CoV-2 Lockdown on Glycaemic Control: A Retrospective Observational Cohort Study in a Tertiary Setting

**DOI:** 10.3390/jcm10184098

**Published:** 2021-09-10

**Authors:** Stefan Zechmann, Leana Hotz, Stefania Di Gangi, Klaus Baumgartl, Andreas Plate, Eliska Potlukova

**Affiliations:** 1 Department of Endocrinology, Diabetes and Metabolism, University Hospital Basel, 4031 Basel, Switzerland; stefan.zechmann@usz.ch; 2 Institute of Primary Care, University and University Hospital Zurich, 8091 Zurich, Switzerland; stefania.digangi@usz.ch (S.D.G.); Andreas.Plate@usz.ch (A.P.); 3 Faculty of Medicine, University of Basel, 4001 Basel, Switzerland; leana.hotz@stud.unibas.ch; 4 Department of Information and Communications Technology, University Hospital Basel, 4031 Basel, Switzerland; klaus.baumgartl@usb.ch; 5 Division of Internal Medicine, University Hospital Basel, 4031 Basel, Switzerland

**Keywords:** COVID-19, SARS-CoV-2, lockdown, glycaemic control, diabetes mellitus, seasonal variation

## Abstract

We analysed the effects of Swiss national lockdown due to the COVID-19 pandemic on the glycaemic control in patients with diabetes mellitus. In a retrospective observational cohort study with observation period 16 December 2018–27 July 2020, we included tertiary care patients with diabetes and at least one glycated haemoglobin A1c (HbA1c) measurement before and after the lockdown beginning. Main outcome measure was change in HbA1c after the lockdown. We included 1078 patients (86% diabetes type 2) with a mean HbA1c of 55.63 mmol/mol (7.24%). Glycaemic control was susceptible to seasonal changes with higher mean HbA1c in winter as compared to spring (57.49 mmol/mol (7.41%) vs. 55.52 mmol/mol (7.23%), *p* = 0.013). The lockdown did not affect the mean HbA1c values of all patients. However, we found a higher proportion of type 2 diabetes patients with a worsening HbA1c after the lockdown as compared to the year before (32% vs. 22.9%, *p* = 0.02). In a mixed-model regression multivariable analysis, inappropriate alcohol intake and hypothyroidism were associated with an increase in HbA1c after the lockdown. In conclusion, the national lockdown had no effect on overall mean HbA1c values but affected a proportion of type 2 diabetes patients with worsening HbA1c, whose individual risk factors were identified.

## 1. Introduction

Natural disasters have been shown to have a long-lasting negative effect on glycaemic control, primarily due to the unavailability of healthcare products and lack of medical care [1,2]. Severe acute respiratory syndrome coronavirus 2 (SARS-CoV-2) pandemic followed by profound societal changes bears features of a global natural disaster. National lockdowns imposed strict restrictions on the populations involved with negative impact on physical activity levels, eating habits, and increased incidence of psychiatric disorders [3,4,5,6,7,8,9,10].

Patients with diabetes are vulnerable to severe forms of coronavirus disease 2019 (COVID-19). In addition, insufficient glycaemic control poses a higher risk for a worse clinical course [11]. It is not clear how nationwide lockdowns influenced the glycaemic control. Several smaller studies with short term follow-up have shown either a positive effect of the lockdown on glycaemic control or no effect at all [12,13,14,15,16,17,18,19,20,21].

We hypothesized that a nationwide lockdown led to a deterioration in glycaemic control in patients with diabetes in the long term. We investigated whether patients with type 1 and 2 diabetes increase their glycated haemoglobin A1c (HbA1c) in response to the Swiss national lockdown as an expression of deterioration in glycaemic control. Furthermore, we aimed to identify the most vulnerable patients to decline in their glycaemic control. In a secondary analysis, we investigated the effect of lockdown on weight and the number of outpatient consultations and hospitalisations in patients with diabetes.

## 2. Materials and Methods

### 2.1. Study Design and Setting

For this retrospective observational cohort analysis study, we used data from electronic health records from all clinics of the University Hospital Basel (USB), one of the five university hospitals in Switzerland and the only tertiary care centre in the north-western part of Switzerland. We defined the observational period as 16 December 2018 until 27 July 2020. Based on the duration of the national lockdown (17 March 2020–26 April 2020), we defined four periods: 3 months before the lockdown (Winter 2019–2020), 4 months starting at the beginning of the lockdown (Spring 2020), and the corresponding periods one year before. In summary, we analysed four periods: (1) Winter 2018–2019 (16 December 2018–16 March 2019), (2) Spring 2019 (17 March 2019–27 July 2019), (3) Winter 2019–2020 (16 December 2019–16 March 2020), and (4) Spring 2020 (17 March 2020–27 July 2020).

### 2.2. Data Extraction

The data system used at the USB was the “Health engine” Version 8.11.5.2 (Netcetera, Zurich, Switzerland) and the “ISMed” Version v19.11_b1 (Protecdata AG, Boswil, Switzerland). We extracted the data via self-developed SQL (structured query language) scripts and “Toad for Oracle” Version 13.2.0.258 (Quest Software Inc., Aliso Viejo, CA, USA). We generated an anonymised dataset without coding and without the possibility of tracing back the anonymised data to the source data.

### 2.3. Participants

We defined as eligible all patients with diabetes who had at least one measurement of HbA1c before and one measurement after the beginning of the lockdown within the observation period, as documented in the hospital database. We defined patients with diabetes as those with a diagnosis of diabetes mellitus or at least one HbA1c measurement of ≥47.55 mmol/mol (6.5%). We stratified the patients according to the type of diabetes (1, 2, other). “Other” diabetes types included Latent Autoimmune Diabetes in Adults (LADA), Maturity Onset Diabetes of the Young (MODY), and pancreatogenic diabetes. We reported results for patients with diabetes type 1 and 2 only.

We excluded patients younger than 18 years and those with documented written or oral refusal to participate in scientific research.

### 2.4. Variables

The main outcome measure was the rate of change of HbA1c value from Winter 2019–2020 to Spring 2020. As potential confounders, we considered comorbidities, history, medication, socioeconomic characteristics, anthropologic characteristics and selected laboratory values.

### 2.5. Definition of Comorbidities

We defined comorbidities according to the diagnosis stated in the hospital database and based on available laboratory or anthropometric values.

The following comorbidities were considered: Hypertension, based on diagnosis and/or systolic blood pressure ≥140 and/or diastolic blood pressure ≥90 mmHg twice documented; overweight, diagnosis and/or BMI (≥25 and <30 kg/m^2^); obesity, diagnosis and/or BMI (≥30 kg/m^2^); hyperlipidaemia, diagnosis and/or low-density lipoprotein ≥4.9 mmol/L; chronic kidney disease, diagnosis and/or glomerular filtration rate (GFR based on CKD-EPI formula ≤90 mL/min); thyroid disease: diagnosis or TSH < 0.25 mIU/L or > 10 mIU/L.

The following comorbidities were based on the stated diagnoses in the database only: obstructive sleep apnoea syndrome, coronary heart disease, chronic heart failure, stroke, periphery artery occlusive disease, diabetic periphery neuropathy, diabetic retinopathy, gout, depression, oncological disease, COPD, asthma, inappropriate alcohol use (documented as such, without quantification).

### 2.6. Other Confounding Factors

We extracted data on all generic and trade names of antidiabetic medication available in Switzerland in the first half of 2020. We divided them into the following: oral antidiabetic drugs (OAD) including metformin, Sodium-Glucose Transport protein 2 (SGLT2)-inhibitors, Dipeptidyl Peptidase-4 (DPP-4)-inhibitors, sulfonylureas, and others (acarbose, glitazones, glinides); insulin short and long-acting; Glucagon-like Peptide-1 (GLP-1) agonists, and their combinations.

For socioeconomic characteristics, we used patient self-reported data documented in the hospital database. We categorized the patients according to their employment (definitions of employment according to British Registrar General’s Scale and adapted in line with Beer-Borst et al. [22]), language, religion, residence, insurance class and whether the patient had a general practitioner. We extracted data on the number of hospitalizations in the observed periods. In patients followed at the Department of Endocrinology, we also raised data on the number of outpatient consultations (due to the billing system to insurance companies, the data on outpatient consultations in other departments were not suitable for analysis).

For the patients’ description at baseline, we used the last available database entry before the beginning of the lockdown. Anthropometric characteristics included age (years) at first HbA1c measurement, gender, weight (kg), BMI (kg/m^2^) and blood pressure (mmHg). Moreover, we also considered smoking habits, defined as yes/no.

### 2.7. Laboratory Methods

For measurement of HbA1c, two methods were used: first, venous HbA1c using turbidimetry (Cobas 8000 modular analyser c502 by Roche Diagnostics International Ltd, Rotkreuz, Switzerland); second, the point-of-care (POCT) analysis using POCT DCA Vantage Analyser (by Siemens Healthcare GmbH, Erlangen, Germany) a point-of-care immunodiagnostic analysis system using monoclonal antibody agglutination reaction. These two methods were being routinely used at the USB. In a sensitivity analysis, the distribution of their values was not statistically different (*p* = 0.98, Kolmogorov–Smirnov test). The proportion of usage of the two methods reached 57% in turbidimetry and 43% in POCT and was stable throughout the years with no significant seasonal changes.

The following parameters were measured using the cobas^®^ 8000 modular analyser (Roche Diagnostics International Ltd, Rotkreuz, Switzerland): glycaemia, low-density-lipoprotein, thyroid-stimulating hormone, creatinine, albumin in the urine.

### 2.8. Biases

To limit potential detection bias, we included only reliable data, and we excluded outliers. To define comorbidities, we used documented diagnoses and laboratory and anthropometric values. Information on current medication was derived from official medical reports and unofficial medical entries in the database. Potential selection bias could occur as two types of HbA1c measurements were used in our hospital. However, sensitivity analysis showed no statistically significant differences in the distribution of values. To tackle the attrition bias, we performed a subanalysis in patients with HbA1c values in all four analysed periods.

### 2.9. Study Size

**Figure 1:**
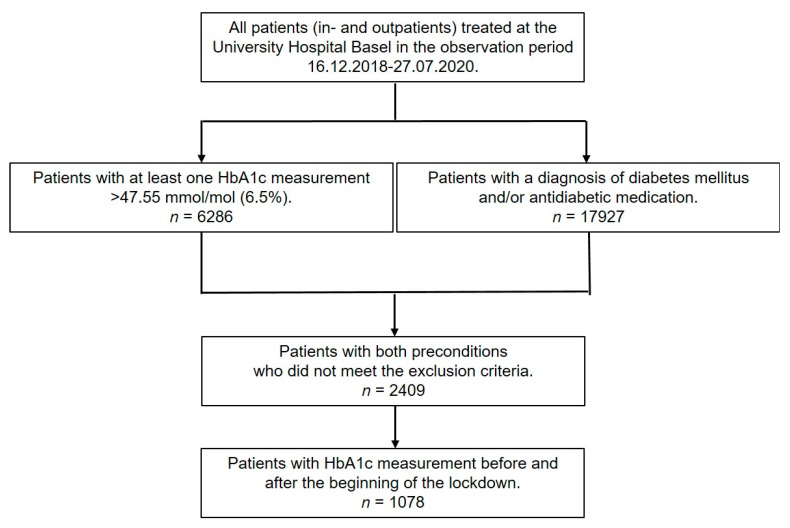
Flow diagram showing the numbers of eligible and included patients.

We included all eligible patients who fulfilled the inclusion criteria in the observation period and who were not younger than 18 years and did not refuse to participate in scientific research (Figure 1).

### 2.10. Statistical Analysis

Patients’ characteristics were reported overall and by diabetes type (1, 2, other). Binary or categorical variables (e.g., sex, comorbidities) were described as a number of cases and percentage of non-missing observations, *N* (%) unless otherwise stated. Continuous variables (e.g., age, weight) were described as mean and standard deviation (SD).

Selected patient characteristics (HbA1c, weight, number of outpatients’ consultations and number of hospitalizations) were compared between all four periods and within the types of diabetes 1 and 2. HbA1c values were reported both in mmol/mol and %.

T-tests for pairwise comparisons of means (Winter 2018–2019 vs. Spring 2019; Winter 2019–2020 vs. Spring 2020) were performed for continuous variables, indicating the mean difference and the 95% (CI). Pearson’s chi-squared test was used to compare proportions of binary variables. Results of these comparisons were finally assessed using mixed regression analysis, adjusting for repeated measurements within patients through random effects. Weekly trends in the four periods of HbA1c values, by the two types of diabetes mellitus, were also modelled with random effects for patients and type of diabetes mellitus and time (in weeks) as fixed effects. We used a three-basis spline model to describe the seasonal pattern of HbA1c over time.

The rate of change of HbA1c value from Winter to Spring was defined as (HbA1c in Spring-HbA1c in Winter)/(HbA1c in Winter). It was calculated for both years (2018–2019 and 2019–2020) at the patient level. A worsening in individual HbA1c value was defined when the rate of change of HbA1c was greater than 5%. An improvement in individual HbA1c value was defined when the rate of change of HbA1c was lesser than −5%.

For the main regression analysis, the outcome of interest was the rate of change of HbA1c value from Winter 2019–2020 to Spring 2020. We performed linear regression models with patients’ characteristics and the overall trend of HbA1c in the previous period (from Winter 2018–2019 to Winter 2019–2020) as predictors. The previous period’s trend was defined as increasing if the average rate of change from Winter 2018–2019 to Winter 2019–2020 was greater than 5%, decreasing if less than −5% and stable if between −5% and 5%. Regression analysis was univariable and multivariable. In the latter case, the effect of a factor was corrected for the effect of the others. Multivariable models were developed starting from variables with *p* ≤ 0.2 in univariable analysis and then implementing a stepwise backward elimination to include all relevant factors which better fit the models. Missing observations were removed from the analysis. Results of regression analysis were reported as estimates (95% CI). For all tests, *p* ≤ 0.05 was considered statistically significant. All analyses were carried out using statistical package R version 4.1.0 (https://www.R-project.org (accessed on 7 September 2021).

## 3. Results

### 3.1. Participants–Descriptive Data

Figure 1 shows the inclusion of patients in the form of a flow diagram. Basic patient characteristics and data on comorbidities and antidiabetic medication are presented in Table 1, where we considered the last available information from the study start to the day before the lockdown. We included 1078 patients (mean age 59.05 years, 63.8% male) in the analysis. Of these, 925 had type 2, 145 type 1 and 8 other forms of diabetes mellitus.

The mean interval between the beginning of the lockdown and the postlockdown HbA1c measurement was 67 days (median 69, min. 0; max 122).

Description of socioeconomic factors of included patients is summarised in Table 2. Most patients pursued a low or manual occupation, or were pensioners. Most lived in an urban region and indicated German as their first language.

Only 6/329 patients tested were positive for SARS-CoV-2 anytime during observation.

### 3.2. Outcome Data

#### Glycaemic Control in the Longitudinal Follow-Up: Seasonal Changes

**Figure 2:**
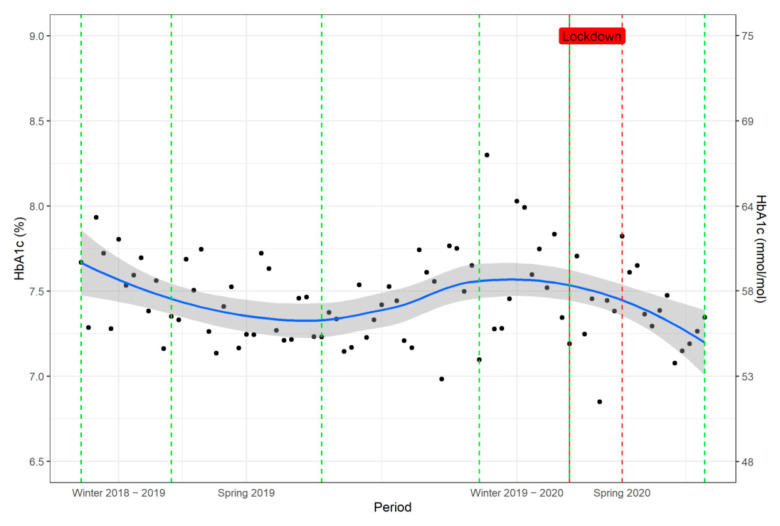
Seasonal variations in HbA1c weekly values in 1078 patients with diabetes mellitus expressed as mean values of HbA1c in observation period 16 December 2018–27 July 2020. The blue line represents the smoothing and the grey field the 95% confidence interval band. Black dots show the mean HbA1c per week. Vertical dashed green lines define the beginning and the end of the individual periods, while the vertical dashed red lines depict the beginning and end of the Swiss national lockdown (the line on the left also defines the beginning of the period Spring 2020).

Throughout the whole observation period, glycaemic control expressed as HbA1c was susceptible to marked seasonal changes (Figure 2). In general, HbA1c increased in winter and decreased in the spring/summer. In all patients, we observed the highest HbA1c values in January with a mean (SD) of 59.57 (18.38) mmol/mol (7.6 (1.68)%), and lowest in July with a mean (SD) of 56.18 (18.27) mmol/mol (7.29 (1.67)%). The estimated absolute difference of HbA1c values between winter and spring, after correcting for repeated measurements, comprised reductions of −2.08 mmol/mol (95% CI: −3.06, −1.09, *p* < 0.001) (−0.19%, 95% CI: −0.28, −0.10) HbA1c in 2019 and −1.31 mmol/mol (95% CI: −2.30, −0.44, *p* = 0.006) (−0.12%, 95% CI: −0.21, −0.04) in 2020.

**Figure 3:**
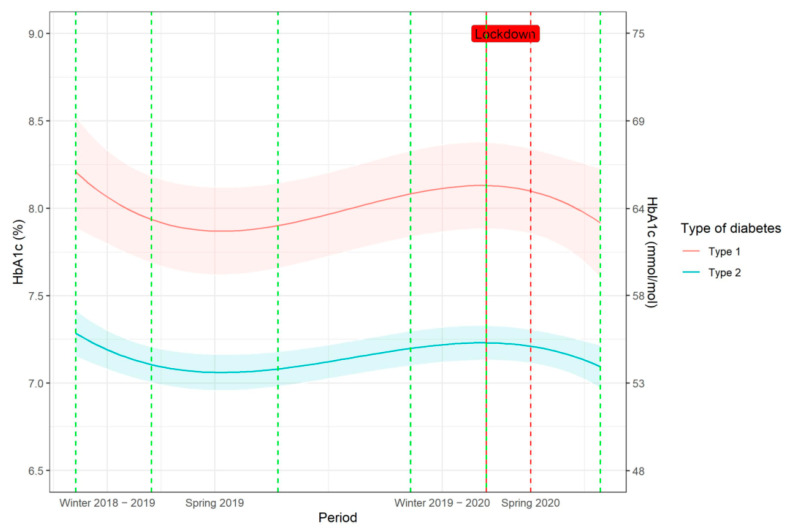
Seasonal variation of HbA1c in patients with type 1 diabetes mellitus (*n* = 145) and type 2 diabetes mellitus (*n* = 925) expressed as fitted values HbA1c in observation period 16 December 2018–27 July 2020 corrected for repeated measurements within patients. The curves represent the fitted values and the coloured fields the 95% confidence interval of the estimates for each diabetes mellitus type. Vertical dashed green lines define the beginning and the end of the individual periods; while the vertical dashed red lines depict the beginning and end of the Swiss national lockdown (the line on the left also defines the beginning of the period spring 2020).

HbA1c values in patients stratified according to the type of diabetes are shown in Figure 3. Patients with type 2 diabetes had the lowest mean HbA1c values in August (54.87 mmol/mol, 7.17%) and in July (54.98 mmol/mol, 7.18%) in 2019 and 2020, respectively; and the highest mean HbA1c values in December (58.69 mmol/mol, 7.52%) and in January (59.24 mmol/mol, 7.57%) in 2019 and 2020, respectively. Patients with type 1 diabetes had higher weekly mean HbA1c values (estimated absolute difference: 10.61 mmol/mol, (95% CI: 5.91, 15.43, *p* < 0.001) (0.97% (95% CI: 0.54, 1.40)), and followed a similar pattern of circannual variation compared to patients with type 2 diabetes.

### 3.3. Main Results

#### Effect of Lockdown on Glycaemic Control

We report the main findings in Table 3. In patients with type 2 diabetes, we found a significantly higher proportion of patients with a worsening in individual HbA1c after the lockdown (a rate of change > 5%). The proportion of patients with an improvement (rate of change HbA1c < −5%) did not differ.

Comparing the changes in mean HbA1c values of type 1 diabetes patients between corresponding winter and spring periods in 2018–2019 and 2019–2020, we observed no statistical differences. We found neither a significantly higher proportion of patients with a worsening in individual HbA1c nor a higher proportion of patients with an improvement of individual HbA1c levels.

Moreover, we also addressed the question of HbA1c values within the period Spring 2020. We found that mean values of HbA1c in Spring 2020 were higher in the 2 months including the lockdown (17 March 2020–16 May 2020) as compared to the following 2 months (17 May 2020–27 July 2020); mean (SD): 7.45 (1.61)% vs. 7.22 (1.59)%, *p* = 0.016.

### 3.4. Additional Analyses

In a sensitivity analysis, a subgroup of 241 patients with either type 1 or type 2 diabetes and at least one HbA1c in every analysed period yielded no significant change in HbA1c after the lockdown compared to the winter period 2019–2020. The HbA1c reached 60.61 mmol/mol (7.7%) in both spring periods.

### 3.5. Effect of Seasonal Variations and Lockdown on Weight

In the 415 patients with available weight values in spring 2020, after correcting for repeated measurements within patients and type of diabetes, we found a significant reduction of weight from winter to spring 2019, with an estimate of −1.16 kg (95% CI: −1.59, −0.72, *p* < 0.001) and from winter to spring 2020, estimate −0.80 kg (95% CI: −1.46, −0.15, *p* = 0.016).

### 3.6. Effect of Seasonal Variation and Lockdown on Outpatient Consultations and Hospitalisations

We did not find any significant seasonal impact on the number of outpatient consultations and hospitalisations in patients with both types of diabetes. However, the total number of both outpatient consultations and hospitalisations per patient and period dropped significantly between Winter 2019–2020 and Spring 2020 (1.83 (SD 1.36) vs. 1.43 (SD 0.93). *p* = 0.001, and 1.42 (SD 0.83) vs. 1.24 (0.55) *p* = 0.028, respectively). In patients with type 2 diabetes, the outpatients’ consultations per patient and period decreased significantly (1.94 (SD 1.45) vs. 1.45 (SD 0.98) *p* < 0.001), while the decrease in hospitalisations was not significant (1.41 (SD 0.83) vs. 1.26 (SD 0.56) *p* = 0.066). In patients with type 1 diabetes, neither hospitalisations nor outpatient consultations changed significantly after the lockdown.

### 3.7. Impact of Confounding Factors on Glycaemic Control after the Lockdown

Univariable regression analysis of the rate of change of HbA1c values from Winter 2019–2020 to Spring 2020 showed a significant impact on HbA1c after lockdown for several variables (Table 4). Type 2 diabetes, oncological disease, treatment with DDP4-inhibitor and GLP-1 agonist, higher LDL levels, speaking a different language than one of the three official Swiss languages and a higher number of hospitalizations and outpatient consultations were associated with an increased HbA1c after the lockdown. In contrast, being overweight and an increase in HbA1c in the previous period were associated with decreased HbA1c after the lockdown.

In the multivariable regression analysis, hypothyroidism, inappropriate alcohol use, treatment with a DPP-4 inhibitor and a combination of GLP-1 agonist with other oral antidiabetic drugs were associated with increased HbA1c after lockdown. A previous increase of HbA1c was associated with an HbA1c reduction after the lockdown (Table 4).

## 4. Discussion

Our study analysed the effect of the national lockdown in spring 2020 on glycaemic control in 925 patients with type 2 diabetes and 145 patients with type 1 diabetes. We found that patients’ HbA1c was susceptible to marked seasonal changes, with HbA1c highest in January and lowest in July. Although the change of mean HbA1c values in both types of diabetes patients did not differ in the current lockdown period compared with the year before, we found a higher proportion of type 2 diabetes patients with a worsening HbA1c after the lockdown. In addition, we could identify hypothyroidism, inappropriate alcohol use, treatment with a DPP-4 inhibitor and a combination of GLP-1 agonist with other oral antidiabetic drugs as individual risk factors associated with an increase of HbA1c after the lockdown.

In contrast to the available studies, we included a large number of patients with diabetes type 2, and we analysed a follow-up period of 4 months after the lockdown beginning. Our cohort is well-described, giving us the opportunity to consider a broad spectrum of variables in an explorative way. Furthermore, we are the first to describe a seasonal variation of HbA1c in central Europe and in the era of new antidiabetic drugs.

Our study has several limitations. First, most patients have only one HbA1c value measurement before, and one after the lockdown beginning, and only a subgroup had HbA1c measurements in all periods. Results in this subgroup did not contradict the main findings. Second, we could not gain information on physical activity and nutrition. Third, we are aware of a selection bias due to studying a population of patients treated at a tertiary care centre. Patients with diabetes treated in the primary care only might differ in their glycaemic control. Fourth, we present data only of 4 months of follow-up after the beginning of the lockdown. Thus, a higher proportion of multimorbid and poorly controlled patients might have been included since the well-controlled patients were followed every 6 months. As the focus of our study was to investigate patients at risk, we do not regard this as a major drawback. We are also aware of a substantial proportion of missing values in the subgroup analyses whose results must be interpreted cautiously.

### 4.1. Effect of Lockdown on Patients with Type 2 Diabetes

Based on the restrictions and negative lifestyle changes imposed by the lockdown, we expected to find a post-lockdown deterioration in glycaemic control, as several studies suggested [3,4,5,6,7,8,9,10,23]. Until now, six smaller studies have analysed the effect of lockdown on glycaemic control in patients with diabetes type 2 with conflicting results, and none took into account the seasonal changes. Our findings are in line with the recent study of D’Onofrio, who did not find a significant impact of lockdown on 141 patients with type 2 diabetes in Italy [21]. Other studies had conflicting results [12,16,17,18,19].

The reasons for these findings are multiple. The assumption that people deteriorated their eating habits during lockdown may be applicable only in selected patient groups and may differ between countries and regions. For example, an Italian study has shown healthier nutrition in the younger population during lockdown [24], and a Finnish study showed an improvement in nutritional habits despite the decreased physical activity [25]. In Switzerland, the overall amount of alcohol sold during lockdown decreased [26], and the consumption of organic food products increased [27].

Moreover, although the population’s mobility dropped dramatically during the lockdown in Switzerland [28], the total physical activity might not have been reduced [29,30]. In Switzerland, no strict rules of staying inside were implemented, and people were free to move outside their homes. More stringent lockdowns with imposed home confinement might lead to different trends in glycaemic control.

### 4.2. Seasonal Variation

Seasons had a strong effect on the glycaemic control: HbA1c increased in winter and decreased in the spring/summer. This effect was strong enough to manifest even in our cohort of patients with good glycaemic control, treated with the newest therapies. We also observed a spring-related decrease of weight of approximately one kilogram in both years analysed. We assume that these seasonal effects are closely linked to increased physical activity during the spring and summer months as compared to winter.

The finding of seasonal variation in glycaemic control is not new, and it is dependent on geographical location. Several studies from Europe and US have reported high HbA1c values in the winter and low in the summer [31,32,33,34,35,36,37,38,39]. An Israeli study showed an inverted relationship between glycaemic control and seasons due to the high temperatures in the summer [40]. We are the first to describe seasonal variation in Central Europe.

### 4.3. Effects of Lockdown on Patients with Type 1 Diabetes

Our study is the first to present data in type 1 diabetes patients in a follow-up of several months after the beginning of the lockdown. The four previously published studies focused on glycaemic control within or immediately after the lockdown showed mostly improved glycaemic control or no change [13,14,15,20]. Our results correspond with these findings and extend them in a longer follow-up.

The glycaemic control of patients with type 1 diabetes was susceptible to similar seasonal variations as patients with type 2 diabetes.

### 4.4. Risk Factors for Deterioration in Glycaemic Compensation after Lockdown

The proportion of patients with type 2 diabetes with a worsening HbA1c was higher in the lockdown period compared to the year before. We identified several individual risk factors associated with an HbA1c worsening. In the multivariable regression analysis, hypothyroidism and inappropriate alcohol use were associated with an increase in HbA1c after the lockdown. We interpret these findings as a negative metabolic effect of hypothyroidism and alcohol use, rendering patients with diabetes more susceptible to adverse changes caused by the lockdown.

Moreover, treatment with DPP-4 inhibitors and GLP-1 analogues combined with any oral antidiabetic drugs was also associated with worsening of the glycaemic control after the lockdown. An explanation is not easy. We suppose that such treatment only expresses a generally worse control of an individual patient, who might be more susceptible to adverse metabolic effects of the lockdown. Unexpectedly, neither overweight nor obesity was associated with an increased HbA1c after the lockdown, possibly being overruled by the seasonal effect.

Deterioration in glycaemic control in the previous period was associated with improved glycaemic control throughout the observation period, mirroring a possible impact of treatment intensification.

The univariable analysis yielded other risk factors, such as oncological disease and foreign mother tongue. However, we regard these results as less reliable than the multivariate analysis, and we interpret them with caution.

As only a negligible number of patients were infected by the SARS-CoV-2 virus, we could not search for a relationship to glycaemic control.

We explain the finding of a reduced number of outpatient consultations and hospitalizations by the official lockdown measures and partly through the generally observed drop in medical consultations due to fear of SARS-Cov-2 infection.

## 5. Conclusions

The national lockdown in Switzerland did not negatively affect the overall glycaemic control in patients with type 1 and 2 diabetes. The strong seasonal effect might have contributed to the maintenance of the glycaemic control during spring. However, a subgroup of patients with type 2 diabetes was at higher risk for worsening their HbA1c levels after the lockdown. We could identify personal characteristics associated with increased odds for deterioration of individual HbA1c. In future lockdowns, worsening of glycaemic control in these patients could be mitigated by optimised care.

## Figures and Tables

**Table 1:** Patients’ Characteristics before the Lockdown.

	Total N (%)	Diabetes Type 2	Diabetes Type 1
N total	1078 *	925	145
Anthropometric parameters			
Age (years) at first HbA1c measurement	59.05 (15.55)	61.36 (14.07)	45.19 (16.85)
Male gender *n* (%)	688 (63.8)	600 (64.9)	82 (56.6)
Weight (kg), *n* = 589	85.90 (21.15)	89.68 (21.16)	71.81 (13.84)
BMI (kg/m^2^), *n* = 587	29.56 (6.63)	30.95 (6.49)	24.34 (3.63)
BP systolic (mm/Hg), *n* = 408	132.97 (18.00)	133.49 (18.42)	131.04 (15.87)
BP diastolic (mm Hg) *n* = 406	81.65 (10.59)	82.23 (10.70)	79.22 (9.62)
Comorbidities *n* (%)	*n* = 1032	*n* = 893	*n* = 131
Hypertension	734 (71.1)	664 (74.4)	67 (51.1)
Overweight	187 (18.1)	157 (17.6)	28 (21.4)
Obesity	393 (38.1)	379 (42.4)	13 (9.9)
Dyslipidaemia	547 (53.0)	500 (56.0)	43 (32.8)
Obstructive sleep apnoea	162 (15.7)	157 (17.6)	5 (3.8)
Coronary heart disease	300 (29.1)	281 (31.5)	18 (13.7)
Chronic heart failure	132 (12.8)	132 (14.8)	0 (0.0)
Stroke	114 (11.0)	101 (11.3)	10 (7.6)
Periphery artery occlusive disease	126 (12.2)	114 (12.8)	11 (8.4)
Chronic nephropathy	735 (71.2)	660 (73.9)	68 (51.9)
Diabetic periphery neuropathy	360 (34.9)	289 (32.4)	69 (52.7)
Diabetic retinopathy	247 (23.9)	189 (21.2)	58 (44.3)
Gout	86 (8.3)	79 (8.8)	7 (5.3)
Thyroid disease	147 (14.2)	125 (14.0)	21 (16.0)
Depression	125 (12.1)	114 (12.8)	11 (8.4)
Oncological disease	238 (23.1)	224 (25.1)	12 (9.2)
COPD and asthma	173 (16.8)	164 (18.4)	8 (6.1)
Smoking	361 (35.0)	318 (35.6)	41 (31.3)
Inappropriate alcohol use	289 (28.0)	257 (28.8)	29 (22.1)
Antidiabetic medication	*n* = 757	*n* = 621	*n* = 128
Metformin	406 (53.6)	402 (64.7)	4 (3.1)
SGLT2-Hemmer	68 (9.0)	68 (11.0)	0 (0.0)
DPP-4-Inhibitoren	221 (29.2)	219 (35.3)	0 (0.0)
Sulfonylureas	41 (5.4)	41 (6.6)	0 (0.0)
other OAD	11 (1.5)	10 (1.6)	0 (0.0)
At least one OAD	484 (63.9)	478 (77.0)	4 (3.1)
2 OAD preparations	166 (21.9)	165 (26.6)	0 (0.0)
3+ OAD preparations	46 (6.1)	46 (7.4)	0 (0.0)
At least one Insulin	486 (64.2)	351 (56.5)	128 (100)
Insulin with OAD	250 (33.0)	245 (39.5)	4 (3.1)
At least one GLP-1 agonist	226 (29.9)	223 (35.9)	2 (1.6)
GLP-1 agonist with any OAD	150 (19.8)	150 (24.2)	0 (0.0)
Insulin + GLP-1 agonist	138 (18.2)	135 (21.7)	2 (1.6)
OAD only	183 (24.2)	182 (29.1)	0 (0.0)
Insulin only	197 (26.0)	70 (11.3)	122 (95.3)
GLP-1 only	37 (4.9)	37 (6.0)	0 (0.0)
Laboratory parameters mean (SD)			
HbA1c (mmol/mol)	55.63 (17.83)	54.10 (17.39)	64.81 (18.05)
(%)	7.24 (1.63)	7.10 (1.59)	8.08 (1.65)
LDL (mmol/L) *n* = 766	2.05 (0.94)	2.06 (0.95)	2.03 (0.81)
GFR (mL/min) *n* = 1033	75.30 (30.93)	72.10 (29.91)	95.93 (28.57)

Values are expressed as mean and SD unless otherwise stated. * 8 patients were stratified to belong neither to diabetes mellitus type 1 nor diabetes mellitus type 2; 3+ means three plus, equal or more than three.

**Table 2:** Socioeconomic Characteristics of the Patients.

	Total *n* (%)	Diabetes Type 2	Diabetes Type 1
*n* total	1078 *	925	145
Employment *n* = 888 †			
High (professional and intermediate occupations)	58 (6.5)	46 (5.9)	12 (11.5)
Medium (non-manual occupations	71 (8.0)	53 (6.8)	17 (16.3)
Low (manual or lower occupations)	370 (41.7)	318 (40.9)	49 (47.1)
Pensioner (including disability pension)	337 (38.0)	321 (41.3)	16 (15.4)
Other	52 (5.9)	40 (5.1)	10 (9.6)
Language			
German	936 (86.8)	791 (85.5)	138 (95.2)
Italian	36 (3.3)	34 (3.7)	2 (1.4)
French	23 (2.1)	23 (2.5)	0 (0.0)
Other	83 (7.7)	77 (8.3)	5 (3.4)
Religion *n* = 623			
Catholic	193 (31.0)	160 (29.7)	33 (40.7)
Protestant	152 (24.4)	126 (23.4)	25 (30.9)
Moslem	118 (18.9)	113 (21.0)	4 (4.9)
Other	160 (25.7)	140 (26.0)	19 (23.5)
Insurance class			
General	1054 (97.8)	901 (97.4)	145 (100.0)
Private/half private	24 (2.2)	24 (2.6)	0 (0.0)
Residence *n* = 1018			
Urban	890 (87.4)	770 (88.3)	113 (81.9)
Rural	54 (5.3)	41 (4.7)	13 (9.4)
Intermediate	74 (7.3)	61 (7.0)	12 (8.7)
General practitioner			
yes (%)	875 (81.2)	743 (80.3)	124 (85.5)

* 8 patients were stratified to belong neither to diabetes type 1 nor diabetes type 2. † Stratification according to British Registrar General’s Scale: high (I and II from the British classification: professional and intermediate professions); medium (III-N: non-manual occupations); and low (III-M, IV and V: manual or lower occupations).

**Table 3:** Summary of the main results: changes in HbA1c values during the observation period.

Type of Diabetes	Spring–Winter 2018–2019	Spring–Winter 2019–2020	*p*
Absolute variations in HbA1c values			
Type 2			
HbA1c% (mean (SD)	−0.13 (0.81)	−0.06 (1.25)	0.350
HbA1c mmol/mol (mean (SD)	−1.42 (8.85)	−0.66 (13.65)	
Type 1			
HbA1c% (mean (SD)	−0.27(0.70)	−0.25 (0.99)	0.880
HbA1c mmol/mol (mean (SD)	−2.95 (7.66)	−2.73 (10.83)	
Patients improved (%): relative HbA1c variation, delta < −5%			
Type 2	30.3%	29.0%,	0.81
Type 1	33.3%	36.6%	0.84
Patient worsened (%): relative HbA1c variations, delta > 5%			
Type 2	22.9%	32.0%	0.02
Type 1	9.8%	16.1%	0.42

Absolute variations are reported as mean differences in HbA1c values (Spring–Winter) in periods 2018–2019 and 2019–2020. Relative changes are described as proportion of patients with an improvement or worsening (relative change delta < −5% or >5%) in HbA1c values.

**Table 4:** Regression Analysis of the Impact of Confounding Factors on the Rate of Change of HbA1c after the Lockdown.

	Univariable Analysis		Multivariable Analysis *N* Patients = 305	
Patient Characteristic	Estimates [95% CI]	*p*-Value	Estimates (95% CI)	*p*-Value
Anthropometric parameters				
Age (years) at baseline	0.0004 (−0.0004, 0.001)	0.327		
Type of Diabetes = DM 2	0.03 (0.002, 0.07)	0.039		
Gender = W	−0.01 (−0.03, 0.01)	0.408		
Weight (kg)	0.0004 (−0.0004, 0.001)	0.309		
BMI (kg/m^2^)	0.00116 (−0.001, 0.0035)	0.336		
Blood pressure (mm/Hg)				
RR systolic	0.000001 (−0.0009, 0.0009)	0.998		
RR diastolic	−0.0015 (−0.0032, 0.00016)	0.076		
Trend in the previous period (ref decreasing < 5%)				
Increasing > 5%	−0.073 (−0.099, −0.0474)	<0.001	−0.08 (−0.11, −0.05)	<0.001
Stable or dec.inc ≤ 5%	−0.004 (−0.041, 0.032)	0.812	−0.02 (−0.06, 0.02)	0.414
Comorbidities (ref = No)				
Hypertension	0.00245 (−0.106, 0.111)	0.965		
Overweight	−0.0403 (−0.0698, −0.0107)	0.008		
Obesity	0.00505 (−0.0199, 0.03)	0.691		
Hyperlipidemia	0.00668 (−0.0175, 0.0309)	0.588		
Obstructive sleep apnea	−0.0137 (−0.0458, 0.0185)	0.405		
Coronary heart disease	0.00176 (−0.0243, 0.0278)	0.894		
Chronic heart failure	0.0167 (−0.021, 0.0544)	0.386		
Stroke	0.0349 (−0.00352, 0.0733)	0.075		
Periphery artery occlusive disease	−0.0218 (−0.0578, 0.0143)	0.236		
Chronic nephropathy	0.0155 (−0.0112, 0.0422)	0.254		
Periphery neuropathy	−0.00936 (−0.0339, 0.0152)	0.454		
Diabetic retinopathy	−0.00628 (−0.0322, 0.0196)	0.634		
Gout	0.0229 (−0.023, 0.0687)	0.328		
Hypothyroidism	0.0236 (−0.0104, 0.0575)	0.173	0.04 (0.01, 0.08)	0.012
Depression	−0.00737 (−0.0435, 0.0287)	0.688		
Oncological disease	0.0493 (0.0208, 0.0778)	0.001		
COPD and asthma	0.0236 (−0.00745, 0.0546)	0.136		
Smoking	−0.00496 (−0.0298, 0.0198)	0.694		
Inappropriate alcohol use	0.022 (−0.00531, 0.0493)	0.114	0.03 (0.00, 0.06)	0.043
Antidiabetic Medication				
OAD group	0.012 (−0.0154, 0.0394)	0.389		
Metformin	−0.00757 (−0.0341, 0.019)	0.576		
SGLT2-Inhibitors	0.0236 (−0.0207, 0.0679)	0.295		
DPP-4- Inhibitors	0.0375 (0.00831, 0.0666)	0.012	0.03 (0.00, 0.06)	0.030
Sulfonylurea	−0.0486 (−0.104, 0.00705)	0.087	−0.06 (−0.14,0.01)	0.095
Other OAD	−0.056 (−0.169, 0.0573)	0.332		
Insulin group	−0.0162 (−0.0446, 0.0121)	0.261		
Insulin with OAD	0.00464 (−0.0233, 0.0326)	0.744		
GLP-1 agonist group	0.0298 (0.000861, 0.0587)	0.044		
GLP-1 agonist with OAD	0.0246 (−0.008, 0.0572)	0.139	0.04 (0.01,0.07)	0.007
Insulin + GLP-1 agonist	0.0197 (−0.0138, 0.0532)	0.248		
OAD only	0.00245 (−0.0296, 0.0345)	0.881		
Insulin only	−0.0244 (−0.0537, 0.0049)	0.102		
GLP-1 only	0.0567 (−0.018, 0.131)	0.137		
Laboratory parameters				
LDL (mmol/L)	0.0151 (0.00126, 0.0289)	0.032		
Triglycerides (mmol/L)	−0.0139 (−0.039, 0.0112)	0.267		
GFR (mL/min)	−0.000227 (−0.0006, 0.00014)	0.226		
Albumin/creatinine ratio in urine	−0.00010 (−0.00026, 0.00006)	0.22		
Socioeconomic characteristics				
Employment (ref = high occupation)				
Medium (non-manual occupations	0.00774 (−0.0572, 0.0727)	0.815		
Low (manual or lower occupations)	0.0314 (−0.0175, 0.0803)	0.207		
Pensioner (including invalidity-pension)	0.0309 (−0.0184, 0.0803)	0.218		
Other	−0.00955 (−0.0818, 0.0627)	0.795		
Language (ref = DE)				
IT	−0.0267 (−0.093, 0.0395)	0.428		
FR	0.00956 (−0.0603, 0.0794)	0.788		
Other	0.0418 (0.0018, 0.0817)	0.041		
Religion (ref = catholic)				
Evangelic	0.0042 (−0.0413, 0.0497)	0.856		
Muslim	−0.00657 (−0.0508, 0.0377)	0.771		
Other	0.0122 (−0.0305, 0.0549)	0.574		
Insurance class				
Private/half private	0.0596 (−0.0171, 0.136)	0.127		
Residence (ref = Urban)				
Intermediate	−0.013058 (−0.064, 0.03825)	0.617		
Rural	0.008459 (−0.047, 0.06390)	0.764		
Outpatient consultations	0.00483 (0.0011, 0.00855)	0.011		
Hospitalizations	0.00686 (0.00139, 0.0123)	0.014		
General practitioner				
No (ref = yes)	−0.012 (−0.0421, 0.018)	0.432		

## Data Availability

Datasets generated during and/or analysed during the current study are not publicly available but are available from the corresponding author on reasonable request.
